# MicroRNA-222 regulates muscle alternative splicing through Rbm24 during differentiation of skeletal muscle cells

**DOI:** 10.1038/cddis.2016.10

**Published:** 2016-02-04

**Authors:** B Cardinali, M Cappella, C Provenzano, J M Garcia-Manteiga, D Lazarevic, D Cittaro, F Martelli, G Falcone

**Affiliations:** 1 Institute of Cell Biology and Neurobiology, National Research Council, Monterotondo Scalo, Rome, Italy; 2 DAHFMO-Unit of Histology and Medical Embryology, Sapienza University of Rome, Rome, Italy; 3 Center For Translational Genomics and Bioinformatics, San Raffaele Scientific Institute, Milan, Italy; 4 Molecular Cardiology Laboratory, Policlinico San Donato-IRCCS, San Donato Milanese, Milan, Italy

## Abstract

A number of microRNAs have been shown to regulate skeletal muscle development and differentiation. MicroRNA-222 is downregulated during myogenic differentiation and its overexpression leads to alteration of muscle differentiation process and specialized structures. By using RNA-induced silencing complex (RISC) pulldown followed by RNA sequencing, combined with *in silico* microRNA target prediction, we have identified two new targets of microRNA-222 involved in the regulation of myogenic differentiation, Ahnak and Rbm24. Specifically, the RNA-binding protein Rbm24 is a major regulator of muscle-specific alternative splicing and its downregulation by microRNA-222 results in defective exon inclusion impairing the production of muscle-specific isoforms of Coro6, Fxr1 and NACA transcripts. Reconstitution of normal levels of Rbm24 in cells overexpressing microRNA-222 rescues muscle-specific splicing. In conclusion, we have identified a new function of microRNA-222 leading to alteration of myogenic differentiation at the level of alternative splicing, and we provide evidence that this effect is mediated by Rbm24 protein.

During skeletal muscle differentiation, gene expression is tightly regulated at both transcriptional and post-transcriptional levels. MicroRNAs (miRNAs) have emerged as key post-transcriptional modulators of gene expression in virtually all biological processes, including myogenesis.^1^ A number of miRNAs have been implicated in myogenesis and muscle disease, some specifically expressed in muscle cells, others ubiquitously expressed.^2, 3^ Two closely related miRNAs, miR-221 and miR-222, were previously shown to be downmodulated during differentiation and to induce a delay in progression of differentiation and alterations of myotube morphology and contractile structures when overexpressed.^4^ MiR-221 and miR-222 are clustered together in both human and mouse genomic *loci*, are evolutionarily conserved among vertebrates, have similar sequences and an identical target binding ‘seed' region, corresponding to positions 2–8, in both humans and mice. They are ubiquitous and have been found abnormally expressed in several tumors.^5^ In muscle cells, miR-221/222 have been shown to target p27 (Cdkn1b),^4^ β1-syntrophin (Sntb1)^6^ and, more recently, MyoD transcription factor.^7^ In addition, an increase in miR-221/222 expression was described in muscles from *mdx* dystrophic mice and in muscle tissues from several human primary muscle disorders,^8, 9^ linking these miRNAs to muscle disease.

MiRNAs are excised from large stem-loop-containing transcripts and then incorporated into RNA-induced silencing complex (RISC), where they silence target transcripts via translational repression and/or mRNA destabilization.^1, 10^ MiRNAs typically bind to target mRNA 3'UTRs containing short stretches of complementarity to the seed region of the miRNA.^11^ MiRNA target sites in mRNA 5'UTRs and coding regions have also been found, although less commonly.^12^ Through this minimal degree of base pairing, miRNAs can potentially regulate many different transcripts within the same cellular pathways in a coordinated fashion. At the same time, however, this poses the question of identifying biologically relevant miRNA–target interactions. Computational approaches for miRNA target prediction are important tools to narrow down the number of putative targets although they tend to overpredict miRNA-binding sites. Overexpression of miRNAs followed by transcriptome analyses is frequently used to identify miRNA–mRNA interactions and their relevance for phenotypic changes. However, this approach presents some limitations, as effects on primary miRNA targets cannot be distinguished from indirect effects on gene expression, and miRNA targets that are regulated solely by translational repression are missed.^13^ Thus, search for 'functional' miRNAs, actually associated to the RISC and engaged in mRNA target modulation, possibly combined with bioinformatic target prediction tools, may prove more useful. Indeed, recent literature reports highlight the important role of the RISC immunoprecipitation (RISC-IP) technique in identifying functionally relevant miRNA targets both in cell culture systems^14, 15^ and in human brain tissue.^16^

In order to identify among the predicted miR-222 targets those specifically involved in skeletal myogenesis, we combined *in silico* target prediction with RISC-IP followed by next-generation sequencing of co-precipitated RNAs^17, 18, 19^ using primary mouse satellite cells (MSC) ectopically expressing miR-222, as myogenic cell model. This approach allowed us to discover and functionally validate a number of miR-222 target transcripts. In particular, we show here that Rbm24, a muscle-specific RNA-binding protein having a major role in regulation of muscle development and differentiation^20, 21, 22, 23^ is a direct target of miR-222 and its inhibition by miR-222 impairs muscle-specific alternative splicing.

## Results

### RNA sequencing and validation of miR-222 targets in skeletal muscle cells

We have developed a target immunopurification method based on the immunoprecipitation of endogenous RISC complexes enriched for miR-222 and its target mRNAs, using antibodies specific for Ago2, a core component of the RISC. A number of preliminary experiments were performed in post-mitotic MSC myocytes to optimize the RISC-IP conditions. The RISC-IP efficiency was checked by western blot analysis of immunoprecipitated Ago2 protein (IP) compared with total Ago2 before IP (input) and to that remaining in the supernatant after IP (super) (Supplementary Figure S1a). Specific enrichment of known miR-222 target mRNAs such as p27 and p57 (Cdkn1c), was assessed by quantitative PCR (qPCR) analysis of RNAs in RISC-IP complexes from miR-222-overexpressing cells (miR-222-IP) compared with RNAs in RISC-IP from control duplexes transfected cells (control-IP) (Supplementary Figure S1b). In order to minimize possible off-target effects on RISC due to excessive overexpression of transfected miR-222 mimics, we set up the miR-222 duplex concentration such to ensure that the specific enrichment of miR-222 on miR-222-IPs was below 10-fold compared with the endogenous miR-222 detected in control-IP, despite being >100 times in the input RNA (Supplementary Figure S2).

**Figure 1:**
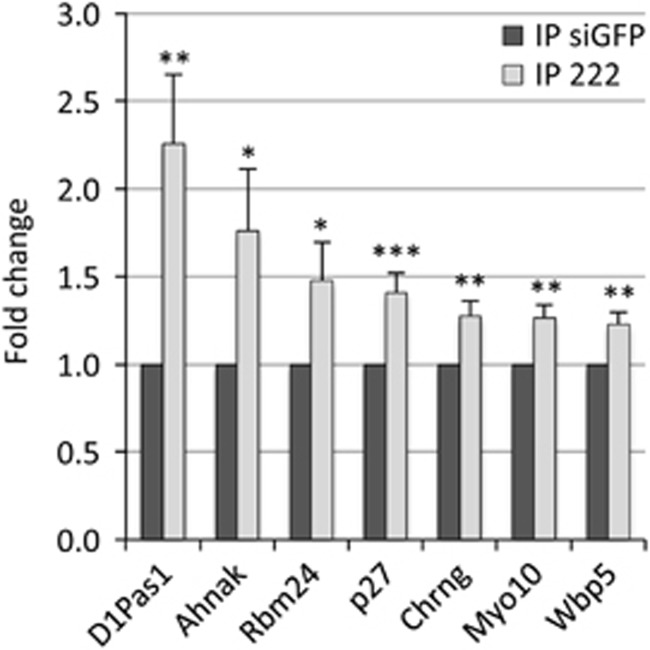
qPCR validation of miR-222 mRNA targets enriched in RISC-IP. MSC myocytes, kept in DM for 30 h, were transfected with 20 nM miR-222 mimic and control siGFP and, after 16 h, processed for RISC-IP. RNA extracted from the IPs were analyzed by qPCR for the enrichment of selected miR-222 mRNA targets. p27 mRNA is included for comparison. Values were normalized to GAPDH mRNA and the expression level of each mRNA is indicated as fold change in miR-222-IPs *versus* control-IPs, referred as 1. The error bars represent the average±S.E. (*n*⩾3–4; *P<0.05; **P<0.01; ***P<0.001)

To identify potential mRNA targets of miR-222, RISC-IP RNAs from MSC myocytes transfected with miR-222 and control duplexes from two independent experiments were analyzed by RNA Sequencing. In this way, a total of 10998 peaks corresponding to 5626 different genes were identified (Supplementary Table S1). The number of peaks exceeded that of the genes as, for some transcripts, more than one peak region was identified, as also previously reported,^16^ in keeping with the fact that a single RNA species can bear more than one miRNA-binding site. Next, putative targets of miR-222 were selected among the RNAs overrepresented in RISC complexes from miR-222-overexpressing cells. To maximize sensitivity and minimize possible biases, a very low cutoff value was adopted (log2 fold change >0). Specificity was ensured by taking into account only transcripts displaying potential miR-222-binding sites and by the following validation step. Using miRanda miRNA target prediction software,^24^ we found that sequence peaks corresponding to 234 different RNAs contained the seed for miR-222 and were overrepresented in miR-222-overexpressing cells (Supplementary Table S2). Among these, six were selected on the basis of their enrichment, gene function and/or relevance for myogenic differentiation, and were validated by qPCR in new RISC-IP experiments (Figure 1 and Table 1). In Figure 1, the average fold increase of the target transcripts in miR-222-IPs compared with control-IPs is shown together with that of the known miR-222 target p27, included for reference. To test whether the increase of these six mRNAs was not simply a reflection of increased total level upon miR-222 expression, RNA extracted from IP input was analyzed. We found that mRNA levels of D1Pas1 and Ahnak were decreased, whereas the other transcripts were not modulated (Supplementary Figure S3). Interestingly, the peak sequences referring to the Chrng gene (Supplementary Table S1) were contained in the 3'UTR of a predicted but not yet verified transcript variant, containing a longer 3'UTR compared with the annotated and sequenced mRNA. We confirmed the existence of such transcript by RT-PCR in both MSC and C2C12 myoblasts (Supplementary Figure S4).

### Ahnak and Rbm24 are novel targets of miR-222 in skeletal muscle cells

Two new potential targets of miR-222 appeared particularly interesting for their pivotal role in myogenic differentiation: Ahnak and Rbm24. Ahnak is a 700 kD large protein belonging to the family of scaffold proteins containing the PDZ protein-interaction domain, involved in regulation of calcium channels, cell architecture and migration and highly expressed in skeletal and cardiac muscle (reviewed in ref. 25). Rbm24 is a 20–25 kD small RNA-binding protein required for cardiac and skeletal muscle development and preferentially expressed in striated muscle where it stabilizes muscle regulatory mRNAs and regulates muscle-specific alternative splicing.^20, 21, 22, 23^

**Figure 2:**
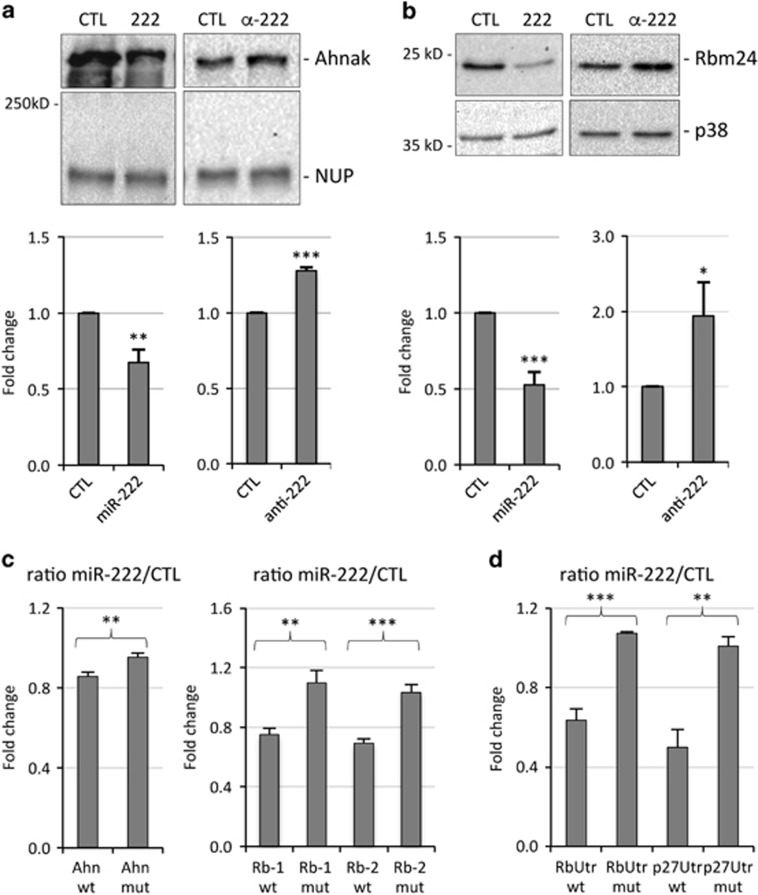
Validation of Ahnak and Rbm24 as direct targets of miR-222. (**a**, **b**) MSC myocytes, kept in DM for 30 h, were transfected with miR-222 mimic (222) or antisense inhibitors (α-222) and relative controls siGFP or anti GFP (CTL). After 48 h, cells were lysed for protein analysis. Western blot analysis of Ahnak (**a**) and Rbm24 (**b**) expression levels; Nucleoporin (NUP) and p38 proteins were used as loading controls. The histograms in (**a**) and (**b**) (lower panels) show the quantitation of expression levels of Ahnak and Rbm24, normalized to Nucleoporin and p38, in myocytes transfected with miR-222 mimic or inhibitor *versus* myocytes transfected with control RNAs, referred as 1. The error bars represent the average±S.E. (*n*=3–6; *P<0.05; **P<0.01; ***P<0.001). (**c**) 293FT cells were transfected with empty firefly luciferase reporter vector (pMIR-REPORT) or derived constructs containing either an intact miR-222-binding site (wt), or a mutated miR-222-binding site (mut) in Ahnak and Rbm24 target regions. For Rbm24 both miR-222 target sites were analyzed (Rb-1, Rb-2). (**d**) 293FT cells were transfected with empty firefly luciferase reporter vector (pGL3) or derived constructs containing an intact portion of the 3'UTR of Rbm24 (RbUtr wt) or p27 ( p27Utr wt) or mutated 3'UTRs (mut). (**c**, **d**) Each plasmid was co-transfected with a plasmid encoding renilla luciferase along with miR-222 mimic or negative control (CTL). Activities of each pMIR-REPORT and pGL3 construct were normalized first to renilla luciferase and then to empty vector activities. The ratio of luciferase activity in cells transfected with miR-222 mimic *versus* cells transfected with control RNA is expressed as fold change. The error bars represent the average±S.E. (*n*=3–5; **P<0.01; ***P<0.001)

To confirm that miR-222 regulates Ahnak and Rbm24 protein expression, we performed experiments of miR-222 overexpression and inhibition in MSC cells. Transfection of myocytes with miR-222 mimic led to a significantly decreased expression of Ahnak and Rbm24 proteins in western blot analysis, whereas transfection with antisense miR-222 inhibitors resulted in the upregulation of Ahnak and Rbm24 protein expression compared with transfected control RNA (Figures 2a and b).

Ahnak mRNA contains a miR-222-binding site in the coding region, whereas Rbm24 mRNA contains two miR-222-binding sites in the 3'UTR (Rb-1 and Rb-2), the most downstream (Rb-2) being that found by RNA sequencing. To show that miR-222 directly regulates the expression of Ahnak and Rbm24 transcripts, miR-222 seed-pairing sites and the immediately surrounding sequences contained in Ahnak and Rbm24 were cloned downstream of the luciferase open-reading frame of the pMIR-REPORT vector. These constructs along with either miR-222 mimic or a control duplex RNA were transfected in 293FT cells and the luciferase activity was measured 24 h later. Figure 2c shows that miR-222 inhibited the expression of the reporter constructs containing an intact miR-222-binding site of Ahnak, whereas this effect was prevented in the construct containing a deletion in the same site. Similarly, both miR-222 seed-pairing sites of Rbm24, but not their mutated versions, were targeted by miR-222. In addition, to analyze the effect of miR-222 on both sites simultaneously, a portion of the 3'UTR of Rbm24 containing both miR-222-binding sites was cloned downstream of the luciferase coding region of pGL3 reporter vector and luciferase expression showed a downmodulation comparable to that of the single sites, and absent in the mutated version. As a reference, the 3'UTR of p27, a well-established miR-222 target^4, 26^ was analyzed, displaying a degree of repression upon miR-222 overexpression similar to that of Rbm24 (Figure 2d). These results confirm that miR-222 directly inhibits Ahnak and Rbm24 target transcripts.

### Overexpression of miR-222 and silencing of Rbm24 result in inhibition of myoblast fusion and muscle alternative splicing

**Figure 3:**
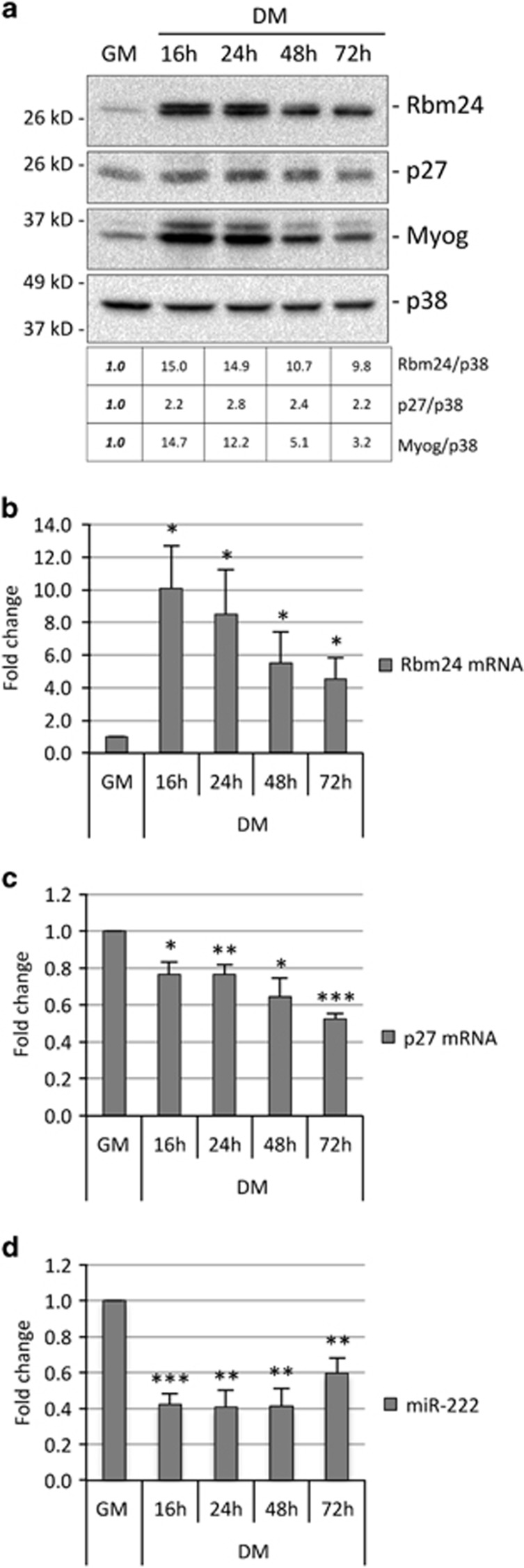
Expression of Rbm24, miR-222 and muscle-specific genes during myogenic differentiation. (**a**) Proliferating MSC myoblasts (GM) and myoblasts shifted to DM for 16–72 h were analyzed at different time points for expression of Rbm24, p27 and Myogenin proteins by western blot. p38 is shown as loadingcontrol. The table shows a quantification of the expression of Rbm24, p27 and Myogenin proteins in DM, normalized to p38, relative to GM, referred as 1. (**b–d**) Parallel cell cultures were analyzed for expression of Rbm24 mRNA, p27 mRNA and miR-222 by qPCR. Values were normalized for expression of GAPDH mRNA and miR-16, respectively, and shown as fold change relative to GM, referred as 1. The error bars represent the average±S.E. (*n=*3–4; *P<0.05; **P<0.01; ***P<0.001)

We chose to focus our studies on Rbm24, as modulation of this protein by miR-222 appears more effective. To verify that Rbm24 is upregulated upon differentiation in MSC, we analyzed Rbm24 expression during differentiation at both mRNA and protein levels, and compared it with that of miR-222. Figure 3 shows the analysis of Rbm24, p27 and muscle-specific Myogenin proteins in growing conditions (growth medium, GM) and at different times following shift to differentiation conditions (differentiation medium, DM), in parallel with Rbm24 and p27 mRNAs, and miR-222, analyzed by qPCR. Myoblasts were plated at low density in GM, as it is known that accumulation of p27 is dependent on cell density in GM.^27^ The results show an inverse expression of Rbm24 or p27 proteins and miR-222 upon differentiation, as expected for miR-222 targets (Figures 3a and d). However, levels of Rbm24 and p27 mRNA show a clear difference upon differentiation, as Rbm24 transcript accumulation was very low in GM compared with DM, whereas that of p27 slightly decreased in DM (Figures 3b and c). This suggests that in myoblasts Rbm24 is regulated mostly at transcriptional level, whereas p27 is repressed posttranscriptionally.

**Figure 4:**
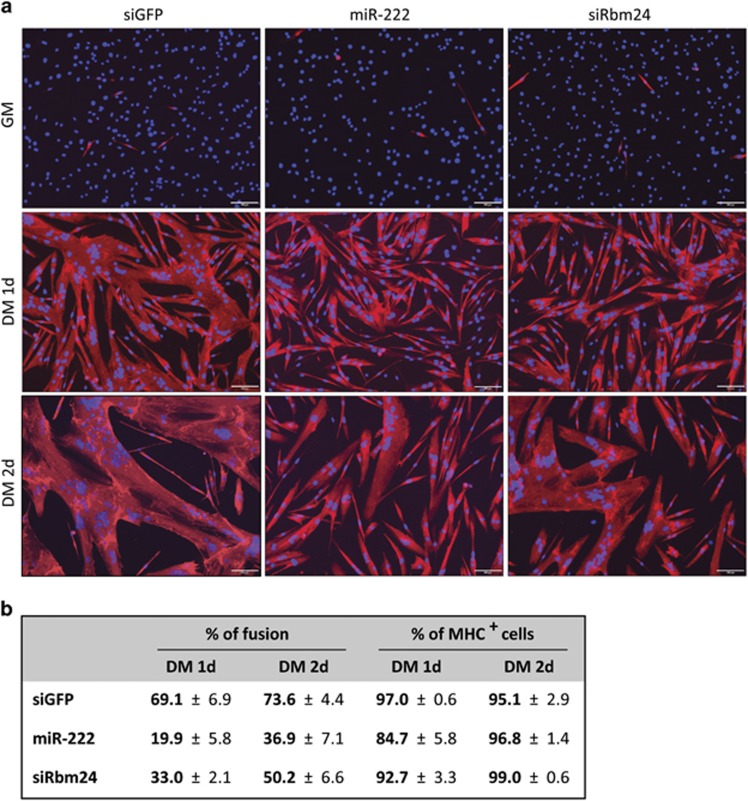
MiR-222 overexpression and Rbm24 silencing inhibit myoblast fusion. (**a**) MSC myoblasts transfected in GM with siGFP, miR-222 or siRbm24 duplex RNAs were maintained in GM for 1 day (GM) or shifted to DM for 1 day (1d) or 2 days (2d). Cells were subjected to immunofluorescence with antibodies specific for skeletal Myosin Heavy Chain (MHC) and nuclei were counterstained with Hoechst dye. Scale bar: 100 *μ*m. (**b**) Quantitation of fusion and MHC-positive (MHC^+^) cells in DM at 1d and 2d in at least three independent experiments (averages±S.E.) is shown in the table. The percentage of fusion was calculated as the ratio of nuclei in MHC^+^ myotubes containing three or more nuclei over total nuclei given as 100% at least 500 nuclei were counted for each plate

**Figure 5:**
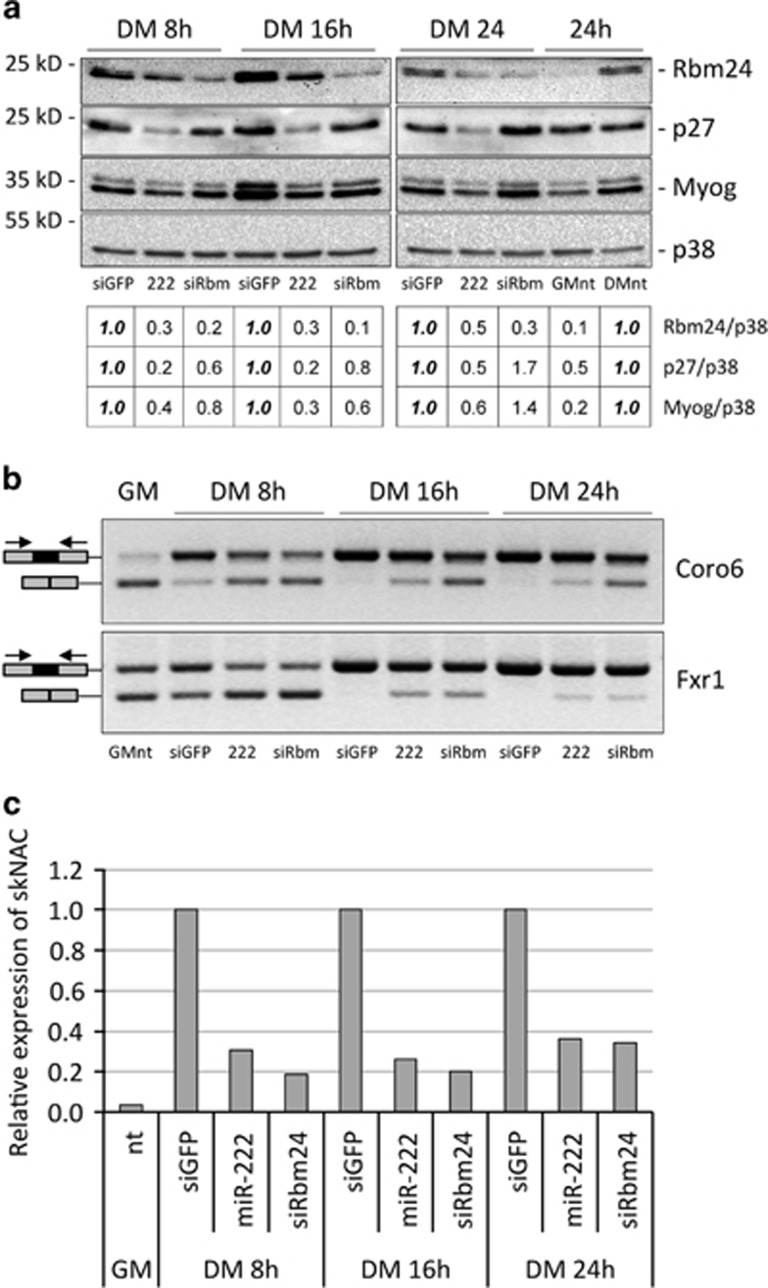
MiR-222 overexpression and Rbm24 silencing inhibit muscle alternative splicing during early myoblasts differentiation. (**a**) MSC myoblasts transfected in GM with siGFP (siGFP), miR-222 (222) or siRbm24 (siRbm) duplex RNAs were shifted to DM for 8 h, 16 h or 24 h and analyzed for expression of Rbm24, p27 and Myogenin proteins by western blot. The table shows a quantification of the expression of Rbm24, p27 and Myogenin proteins normalized to p38, relative to control siGFP, referred as 1. Untransfected MSC in GM (GMnt) and DM (DMnt) at 24 h are shown for comparison. (**b**) RNA from parallel cell cultures was analyzed by semi-quantitative RT-PCR and amplicons were separated on ethidium bromide-stained agarose gels to determine splicing efficiency of muscular isoforms of Coro6 and Fxr1 transcripts. Greyscale of images was inverted for a sharper band definition. In the scheme, the black rectangles represent muscle-specific exons and the black arrows indicate primer positions. (**c**) General and muscular isoforms of NACA transcripts were detected by qPCR analysis and normalized to GAPDH transcript. Expression of muscle-specific isoform of NACA (skNAC) over the general NACA isoform, and relative to siGFP, referred as 1, is shown in the histogram. A representative experiment is shown

In order to determine whether downregulation of Rbm24 contributed to miR-222-inhibition of myogenesis, we decided to investigate whether overexpression of miR-222 and inhibition of Rbm24 by RNAi could exert similar effects on differentiation. To this aim, we transfected miR-222, siRbm24 or control duplex RNAs in proliferating myoblasts, allowed them to differentiate and analyzed the percentage of fusion and the number of myosin-positive cells. Silencing of Rbm24 by RNAi or by miR-222 overexpression resulted in a similar impairment of myoblast fusion, whereas the number of myosin-positive cells was only slightly affected by both treatments (Figure 4). Inhibition of Rbm24 protein accumulation by miR-222 and siRbm24 was verified by western blot after 24 h in DM in a parallel experiment (Figure 5a).

As Rbm24 has an important role in regulation of muscle alternative splicing of several transcripts during skeletal muscle differentiation,^23^ we asked whether miR-222 overexpression could also affect muscle-specific splicing. MSC myoblasts were transfected with miR-222, siRbm24 or control duplex RNAs, shifted to differentiation conditions and, after 8, 16 and 24 h, cells were subjected to western blot analysis to check downregulation of Rbm24 protein and to PCR analysis to measure the efficiency of muscle alternative splicing of mRNAs coding for Coro6, Fxr1 and NACA. These proteins have important roles in skeletal muscle formation and regeneration^28, 29, 30^ and the processing of their transcripts was shown to be regulated by Rbm24 during skeletal muscle differentiation.^23^ Overexpression of miR-222 inhibited efficiently accumulation of Rbm24 and p27 proteins already at 8 h post differentiation, whereas siRbm24 specifically inhibited only Rbm24 protein accumulation, best at 16 h and 24 h post differentiation. Inhibition of Myogenin accumulation was also observed in miR-222 overexpressing cells, possibly as an indirect consequence of miR-222 effect on other targets (Figure 5a). Exon inclusion yielding the muscle-specific isoforms of Coro6, Fxr1 and NACA mRNAs was inhibited by both miR-222 overexpression and Rbm24 RNAi with comparable efficiency. Indeed, increased accumulation of the general isoforms and decrease of the muscle-specific isoforms are well detectable at 16 h and 24 h for Coro6 and Fxr1, and already at 8 h for skNAC, as compared with cells transfected with control duplex RNA (Figures 5b and c).

In order to determine whether inhibition of p27 by miR-222 could also be involved in regulation of Rbm24 expression and, in turn, of myoblast fusion and muscle alternative splicing, we analyzed the effects of miR-222 overexpression and p27 inhibition by RNAi in parallel. These experiments demonstrated that inhibition of p27 did not affect accumulation of Rbm24 protein and, conversely, Rbm24 RNAi did not alter p27 expression level (Supplementary Figure S5c). Moreover, in contrast to miR-222 overexpression and siRbm24, neither myoblast fusion nor muscle alternative splicing were affected by p27 RNAi (Supplementary Figure S5a, b and d).

These results clearly show that decreased accumulation of Rbm24 by means of either miR-222 overexpression or Rbm24 RNAi results in comparable inhibition of myoblasts fusion and muscle-specific alternative splicing, suggesting that the inhibitory effect of miR-222 is mediated by modulation of Rbm24.

### Ectopic expression of Rbm24 rescues miR-222-dependent inhibition of muscle alternative splicing

**Figure 6:**
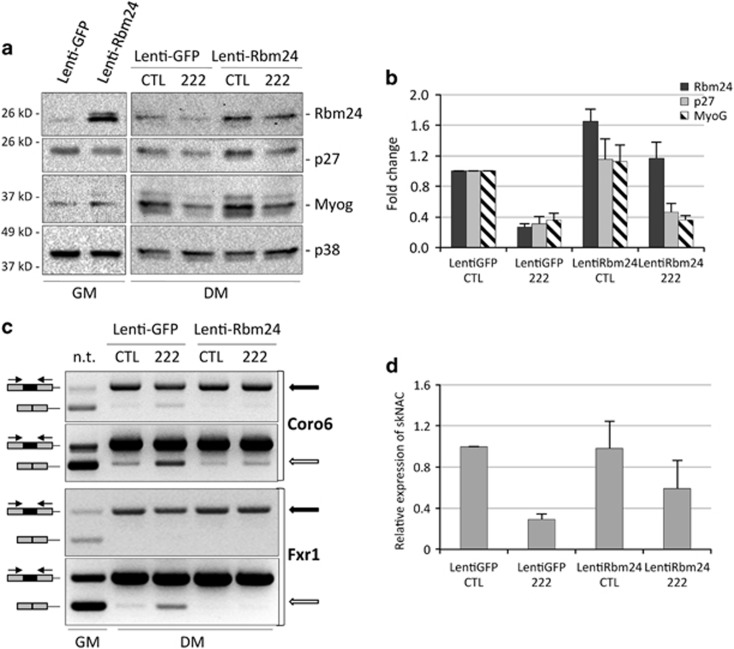
Ectopic expression of Rbm24 rescues muscle alternative splicing inhibited by miR-222. (**a**) MSC myoblasts were infected with Lenti-GFP and Lenti-Rbm24 and grown in GM for 48 h (GM). Parallel cultures were transfected with miR-222 mimic (222) and negative control duplex RNA (CTL) and shifted to DM for 24 h. Cells were analyzed for expression of Rbm24, p27 and Myogenin proteins by western blot. (**b**) The histogram shows the quantitation of the expression levels of Rbm24, p27 and Myogenin normalized to p38, relative to MSC infected with Lenti-GFP and transfected with control duplex RNA, referred as 1, in three independent experiments. The error bars represent the average±S.E. (**c**) RNAs from parallel cell cultures were analyzed by semi-quantitative RT-PCR, and amplicons were separated on ethidium bromide-stained agarose gels to determine splicing efficiency of muscular isoforms of Coro6 and Fxr1 transcripts. Two different exposures of the same amplified bands for Coro6 or amplification at different PCR cycles for Frx1 are shown, to better highlight differences in the muscle-specific isoforms (top panels, black arrows) and in the general isoforms (bottom panels, white arrows). (**d**) Real-time PCR analysis to detect general and muscular isoforms of NACA transcripts normalized to GAPDH transcript. The histogram shows expression of muscle-specific isoform of NACA (skNAC) over the general isoform, and relative to Lenti-GFP transfected with control duplex RNA, referred as 1, in three independent experiments. The error bars represent the average±S.E.

To assess the specific contribution of Rbm24 to the effect of miR-222 on muscle alternative splicing, we generated a lentiviral vector containing the coding region of Rbm24 (Lenti-Rbm24). The cloned allele lacked the 3'UTR region and therefore was unresponsive to miR-222 regulation. Proliferating myoblasts were infected with Lenti-Rbm24 or a control Lenti-GFP (green fluorescent protein), maintained in GM for 48 h to allow for protein expression. Then, cells were either processed for analysis of exogenous Rbm24 protein accumulation or transfected with miR-222 or control duplex RNAs and shifted to DM for 24 h to test for splicing efficiency. Western blot analysis confirmed ectopic expression of Rbm24 protein in GM, where the endogenous protein is barely expressed, and in DM, where its expression adds up to the endogenous form (Figures 6a and b). In miR-222 mimic-transfected cells, lentiviral expression of ectopic Rbm24 reconstituted the physiological levels of the protein, as expression from the endogenous transcript is inhibited (Figures 6a and b). The rescued expression of Rbm24 resulted in increased accumulation of the muscle-specific isoforms of Coro6, Fxr1 and NACA transcripts (Figures 6c and d), confirming that miR-222 inhibits exon inclusion through downregulation of Rbm24. Interestingly, in the presence of miR-222, ectopic Rbm24 did not recover the expression of p27, as expected, being p27 a direct miR-222 target, and did not induce a significant increase in Myogenin accumulation (Figures 6a and b), indicating that regulation of this protein was not dependent on Rbm24 in this context.

## Discussion

The identification of biologically important targets is crucial for understanding miRNA function. *In silico* miRNA target prediction tools combined with biochemical pulldown techniques such as RISC-IP, followed by RNA sequencing, provide a specific and sensitive method for identifying miRNA targets. However, one potential limitation is represented by the low efficiency of the current bioinformatic algorithms to identify non canonical, 'seedless' targets.^31^ Most studies on miR-222 targets have focused on cancer-related pathways, being this miRNA deregulated in many cancer types.^5^ In this study, we searched for miR-222 targets in skeletal muscle cells to identify miR-222-regulated pathways in myogenesis, and assuming that muscle-specific targets could be identified only in a myogenic cell context.

By using this approach, we have been able to identify and validate by qPCR a number of miR-222 target transcripts, two of which were further studied for their crucial roles on skeletal myogenesis. Ahnak contains a miR-222 target sequence in the coding region. There are many examples of transcripts containing miRNA target sites in the coding region, which are translationally regulated.^12^ It is hypothesized that these sites may be recruited when the 3'UTR of the target gene is too short or to tune the protein abundance of alternative splice variants.^12^ We have shown that Ahnak target site is responsive to miR-222 in luciferase reporter assays and that Ahnak protein is modulated following miR-222 overexpression and inhibition in muscle cells. Further experiments are needed to define how downregulation of Ahnak by miR-222 impacts on muscle differentiation. Similarly, we have shown that both miR-222-binding sites of Rbm24 are responsive to miR-222 in luciferase reporter assays, either separately or in combination, and that the protein is modulated by miR-222 overexpression and inhibition. However, Rbm24 transcript stability is not affected by overexpression of miR-222, implying that this target would have not been identified by using a transcriptome analysis approach. As Rbm24 mRNA and miR-222 are inversely regulated upon differentiation of muscle cells, it is likely that miR-222 contributes to ensure a low expression level of the protein in proliferating myoblasts. Notably, upon differentiation Rbm24 transcript is highly upregulated, in contrast to p27. This suggests that Rbm24 is mostly transcriptionally regulated, whereas p27 is translationally repressed in myoblasts.

We show here that overexpression of miR-222 and silencing of Rbm24 result in inhibition of myoblast fusion. Rbm24 silencing was previously shown to inhibit fusion of C2C12 myoblasts undergoing differentiation^20^ and we observe a similar effect in MSC, confirming that Rbm24 is required for myoblast fusion. However, inhibition of myotube formation by miR-222 does not appear to be mediated solely by Rbm24, as ectopic expression of the protein does not result in enhanced fusion (data not shown). As previously reported in other myogenic contexts,^4, 7^ overexpression of miR-222 results in inhibition of Myogenin accumulation also in MSC. However, in contrast to a previous report in C2C12 myoblasts,^21^ in MSC we do not observe a modulation in Myogenin expression following Rbm24 silencing or ectopic expression in the presence of miR-222 mimics. This is not surprising as it is well known that miRNAs affects many targets at the same time, and it is presumable that miR-222 inhibitory effect on Myogenin expression is mediated by other targets such as MyoD.^7^

As it was previously shown that downregulation of p27 affects myoblast fusion and myosin accumulation,^27^ we asked whether p27 could regulate Rbm24 expression and found that p27 silencing neither alters Rbm24 protein accumulation nor myoblast fusion and muscle-specific alternative splicing, implying that the two proteins have different roles in regulation of differentiation. These contrasting results on the effects of p27 can possibly be explained by the use of a different cell model (C2C12 immortalized myoblasts) and different experimental conditions (transfection at high cell density).

It has been reported that skeletal muscle is one of the tissues with the highest number of alternative splicing events, required for the expression of muscle-specific protein isoforms essential for muscle development and function.^32, 33^ Some forms of muscle disease are associated to aberrant alternative splicing. In particular, in Myotonic Dystrophies triplet expansion within non-coding region of RNAs alters either the activity or the expression of the splicing regulators MBNL1 and CUGBP1^32^ and genome-wide analysis of muscle biopsies from Myotonic Dystrophy type 2-affected patients revealed hundreds of aberrant alternative splicing events.^34^ Notably, miR-222 expression levels were found increased in several muscular dystrophies.^8^ Here, we show that targeting Rbm24 by miR-222 is a crucial event in inhibition of muscle-specific transcript production. It is likely that, in addition to Coro6, Frx1 and NACA, miR-222 can regulate the expression of other muscle-specific proteins, important for muscle development and function, through modulation of Rbm24 expression during differentiation. Regulation of alternative splicing by miRNAs during muscle development was previously reported for muscle-specific miR-133. In contrast to miR-222, miR-133 is upregulated during myogenic differentiation and was shown to repress polypyrimidine tract-binding protein (PTB), an inhibitor of early muscle-specific splicing.^35^ Interestingly, PTB antagonizes Rbm24 in promoting muscle-specific exon inclusion.^23^

The number of miR-222 targets involved in skeletal muscle cell differentiation is constantly increasing, and we describe here two new ones. All miR-222 validated targets have important functions in many processes required for myogenesis such as lineage determination and mainteinance (MyoD^7^), control of proliferation (p27^4^), regulation of dystrophin complexes and costameres (β1-syntrophin;^6^ Ahnak, this work) and regulation of muscle-specific alternative splicing (Rbm24, this work). Importantly, we have identified a new function of microRNA-222 leading to alteration of myogenic differentiation at the level of alternative splicing, and we show that this effect is mediated by Rbm24 protein.

These findings altogether highlight the important role of miR-222 in controlling myogenesis. The number of muscle-related miR-222 targets is going to increase in the next future and our list of overrepresented RNAs in RISC-IP from miR-222-overexpressing myocytes is a source of other miR-222 potential targets, which may be relevant for regulation of myogenesis.

## Materials and Methods

### Antibodies

Mouse monoclonal antibody (mAb) anti-Ago2 (MA2) was a gift from Dr. O'Carrol. mAb to p27 was from BD Transduction Laboratories (Franklin Lakes, NJ, USA), and rabbit polyclonal Ab to p38 (C-20) was from Santa Cruz Biotechnology (Santa Cruz, CA, USA). mAbs to Nucleoporin 153 (QE5) and to Ahnak (EM-09) and rabbit polyclonal Ab to RBM24 (94567) were from Abcam (Cambridge, UK). mAb to Myosin Heavy Chain (MF20) was obtained from Dr. Fischman and mAb to myogenin (F5D) was a gift from G Cossu. TRITC-conjugated goat anti-mouse antibodies were from Jackson ImmunoResearch Laboratories (West Grove, PA, USA). Horseradish peroxidase-conjugated goat anti-mouse and anti-rabbit antibodies were from Santa Cruz Biotechnology.

### miRNA mimics, siRNAs and antisense oligonucleotides

miR-222 and control MirVana mimics were hsa-miR-222-3p and Negative Control #1 from Ambion, Thermo Scientific (Waltham, MA, USA). Alternatively, siRNA-like miR-222 mimic and siGFP control RNA duplex (Eurofins, Luxembourg, Luxembourg, described in^4^) were used. 2′-O-Me antisense RNA oligonucleotides anti miR-222 and anti GFP as control were purchased from Eurofins,^4^ whereas siRNA for Rbm24 was Mission Pre-designed siRNA (PDSIRNA2D) from SIGMA-ALDRICH (St. Louis, MO, USA). The antisense sequence of the Individual siGenome duplex (Dharmacon, Lafayette, CO, USA) for mouse p27 was 5′-UAUCCCGGCAGUGCUUCUCUU-3′.

### Plasmid construction

To prepare the Rbm24 lentiviral construct, the coding region of Rbm24 (RefSeq NM_001081425) was amplified by RT-PCR from total RNA of differentiated MSC using the primers described in Supplementary Table S3, also containing the *Age*I and *Sal*I restriction sites. The PCR product was inserted into pCR2.1 (TA Cloning Kit, Thermo Scientific), excised by AgeI and SalI digestion and then cloned into the AgeI and SalI sites of pCCLsin.PPT.hPGK.GFP,^36^ here named Lenti-GFP, by replacing GFP.

To construct the luciferase reporter plasmids (pMIR-Ahn wt, pMIR-Ahn mut, pMIR-Rbm-1 wt, pMIR-Rbm-2 wt, pMIR-Rbm-1 mut, pMIR-Rbm-2 mut), synthetic oligos (Supplementary Table S3) corresponding to 58 nt surrounding the predicted miR-222 target sites (wt) or the same sequence deleted of the 7 nt matching the miR-222 seed sequence (mut) of Ahnak (RefSeq NM_009643.2) and Rbm24 (RefSeq NM_001081425), were cloned into the *Spe*I and *Hin*dIII restriction sites of the pMIR-REPORT Luciferase vector (Ambion, Thermo Scientific). To generate the Rbm24-3'UTR luciferase reporter plasmid, a 959 bp fragment containing two putative miR-222-binding sites was amplified by PCR from differentiated MSC cDNA, using the primers described in Supplementary Table S2. Following SpeI digestion, the PCR product was cloned downstream of the firefly luciferase coding sequence in the pGL3-Promoter vector (Promega, Fitchburg, WI, USA) into the *Xba*I restriction site. The Rbm24-3'UTR fragment was cloned in sense (pRbm-UTR wt) and antisense orientation (pRbm-UTR mut).

### Cell cultures, duplex RNA transfections and viral infections

Primary MSC^37^ were grown in Ham's medium supplemented with 20% fetal bovine serum, 3% chicken embryo extract and 2.5 ng/ml basic fibroblast growth factor on collagen-coated dishes. C2C12^38^ were maintained proliferating in Dulbecco's Modified Eagle medium (DMEM) supplemented with 20% fetal bovine serum (GM). Differentiation of MSC and C2C12 myoblasts was induced by incubating the cultures in DMEM supplemented with 2% heat-inactivated horse serum (DM). MSC myoblasts were transfected with 10–25 nM duplex RNAs (siRNA or miR-222 mimics and controls) or 25–50 nM 2′-O-Methyl antisense RNA oligonucleotides using the Lipofectamine 2000 transfection reagent (Invitrogen, Thermo Scientific, Carlsbad, CA, USA) in serum-free Optimem (Gibco-BRL, Thermo Scientific, USA). MSC myoblasts were transfected either in GM for 4–5 h and transferred to DM for different times, or shifted to DM for 24–30 h and then transfected for 5 h to overnight. 293FT cells (Invitrogen, Thermo Scientific) were grown in DMEM supplemented with 10% fetal bovine serum and used for both lentivirus production and luciferase assays. For production of Lenti-GFP^36^ and Lenti-Rbm24, 5 × 10^6^ cells per 100-mm plate were plated and, the following day, transfected with the lentiviral construct plasmids and packaging mix plasmids (pMD2.VSVG,^39^ pMDLg/pRRE and pRSV-Rev^36^) using the Lipofectamine 2000 reagent. Virus containing supernatant was harvested 48 h and 72 h later and used to infect MSC cells (1 ml per 35-mm plate) along with 2 *μ*g/ml of Polybrene. Virus expression was monitored by western blot analysis of GFP and Rbm24 proteins.

### Ago2 immunoprecipitation

MSC myocytes kept in DM for 30 h were transfected with 20 nM miR-222 or siGFP duplex RNAs using Lipofectamine 2000 reagent (Invitrogen, Thermo Scientific) and maintained in DM for a further 16 h. After cell lysis, two lysate aliquotes were taken to analyze total RNAs and proteins and the remaining was immunoprecipitated with anti-Ago2 antibody using the RNA-binding protein immunoprecipitation Magna RIP Kit (Merck Millipore, Billerica, MA, USA) following the manufacturer's instructions. Ago2 bound RNA was extracted using TRIzol reagent (Invitrogen, Thermo Scientific).

### RNA sequencing

Amplification of cDNA from Ago2 co-immunoprecipitated RNA was performed using the Ovation RNA-Seq system V2 (Nugen, San Carlos, CA, USA), then cDNA was fragmented and ligated into a sequencing library using Ovation Ultralow library system (Nugen). After barcoding, the RNA libraries were pooled, denatured and diluted to a 8 pM final concentration. Cluster formation was performed on cBot (Illumina, San Diego, CA, USA) (paired-end) using flow cells v.3. The SBS (sequencing by synthesis) was performed according to TruSeq PE protocol (Illumina) for the HiSeq 2500 (Illumina) set to 2 × 100 cycles, yielding an average of 30 × 10^6^ clusters for each sample. RNA sequencing data are archived in the Gene Expression Omnibus database (GEO entry GSE75981, http://www.ncbi.nlm.nih.gov/geo/query/acc.cgi?acc=GSE75981). Analysis of count data was performed using htseq-count (v0.5.3p9) and DESeq2^40^ after reads were aligned to the mouse genome (mm10) using SOAPSplice.^41^ In brief, DESeq2 uses generalized linear models based in the negative binomial distribution. We built linear models using two different experiments and two conditions (miR-222 overexpression (*n*=1) and control siRNA (*n*=2)) with the linear formula (~condition+experiment).

### Detection of putative targets

In order to find putative miR-222 seeds within target mRNAs immunoprecipitated with RISC complexes, we identified RNA-Seq enrichments using a peak calling approach. In brief, all aligned reads (bam format) were transformed into bed files using bedtools (version 2.17.0) and peaks were called using macs2 with default parameters. Peaks were filtered by a log_10_p.value>20, in order to enforce trustable enrichments. Peaks coming from the different samples were merged in a single bed file containing all peaks. They were then annotated using the mm10 bed annotation file from Ensembl (Mus_musculus.GRCm38.72.gtf). Read counts over the identified intervals were assessed using bedtools. miRanda software for microRNA target prediction^42^ was used to search for miR-222 putative targets on the peak sequences detected at RISC-IP RNA. Genes with peaks overrepresented in miR-222-overexpressing cells (log2 Fold Change >0) in two independent experiments with miRanda detected seeds for miR-222 were considered for further validation.

### RNA expression analysis

Total and immunoprecipitated RNAs were extracted with TRIzol reagent (Invitrogen, Thermo Scientific) and retro-transcribed with the GoScript Reverse Transcription System (Promega) using oligo (dT) and random primers. Specific primers for mRNA analysis by PCR are shown in Supplementary Table S3. qPCR was performed using an Applied Biosystem 7500 Fast Real-Time PCR System. Power SYBR Green PCR master mix (Applied Biosystems, Thermo Scientific, Waltham, MA, USA) was used to analyze mRNAs and quantification was based on the standard curve method. Results were normalized with respect to GAPDH expression. The analysis of different isoforms of alternatively spliced mRNAs was performed by RT-PCR using GoTaq Flexi DNA Polymerase (Promega) for 25–30 cycles for Coro6 and 30–35 cycles for Fxr1.

microRNA levels were analyzed from total RNA using the TaqMan MicroRNA Assays (Applied Biosystems, Thermo Scientific). Relative expression was calculated using the comparative Ct method (2^−ΔΔCt^).^43^ Different samples were normalized to miR-16 expression.

### Luciferase assays

For the expression of luciferase reporter constructs, 7 × 10^4^ 293FT cells were plated on Poly-L-Lysine (20 *μ*g/ml, L7240 Biochrom, Berlin, Germany) coated 48-multiwell plates and transfected with 7.5 ng of pMIR-Report or pGL3-Promoter and derived plasmids, along with 7.5 ng of pRL plasmid coding for renilla luciferase (Promega) to normalize for transfection efficiency (pRL-CMV in co-trasfection with pMIR, pRL-SV40 in co-trasfection with pGL3). In total, 0.4 *μ*l/well of the lipofectamine 2000 reagent were used (Invitrogen, Thermo Scientific). In some experiments, a pGL3 construct containing the human p27 3'UTR, obtained by A Fusco^26^ was used as positive control. In total, 50 nM of duplex RNAs were co-transfected with the reporter plasmid DNA. After 24 h, cells were lysed and luciferase expression was measured with the Dual Luciferase Assay kit (Promega) using a luminescence counter (1420 Victor Light, PerkinElmer, Waltham, MA, USA).

### Immunofluorescence analysis

For labeling with mAb to myosin heavy chain (MF20), cultures were fixed with a mixture of 3.5% formaldehyde, 70% ethanol and 5% acetic acid and washed thoroughly before incubation with antibody. The samples were examined with an Olympus AX70 immunofluorescence microscope. Images were recorded on an Olympus XM10 camera and processed using the Olympus CellSens Standard 1.8.1 software. Cell scoring was carried out using the public domain software ImageJ.

### Whole-cell extracts and western blot analysis

Cells were lysed in RIPA buffer (140 mM NaCl, 3 mM MgCl2, 1 mM EDTA, 15 mM Hepes, pH 7.2, also containing 0.5% sodium deoxycholate, 1% NP-40, 0.1% SDS) supplemented with a cocktail of protease inhibitors. Western blots were carried out using horseradish peroxidase-conjugated goat anti-rabbit and anti-mouse antibodies and revealed with a chemiluminescence detection system by Thermo Scientific. Imaging and quantitation of the bands were carried out by ChemiDoc XRS Western Blot Imagin System using the ImageLab 4.0 software (Bio-Rad, Hercules, CA, USA).

### Statistical analysis

Variables were analyzed by Student's *t*-test and a probability value of *P*⩽0.05 was deemed statistically significant. Values are expressed as average±S.E.

## Figures and Tables

**Table 1:** Validated miR-222 target transcripts

**Gene symbol**	**RefSeq**	**Average log2 fold change**	**Target sequence position**	**Known functions**^a^
Ahnak	NM_009643	0.45	Coding	Regulation of actin cytoskeleton, calcium channels and skeletal muscle costameres
Chrng	XM_006529078	0.32	3'UTR	Cholinergic receptor activity, protein binding
D1Pas1	NM_033077	0.51	3'UTR	ATP binding, DNA/RNA binding
Myo10	NM_019472	0.26	3'UTR	Actin binding, ATP binding, regulation of cell adhesion and migration
Rbm24	NM_001081425	0.23	3'UTR	RNA binding, regulation of mRNA stability, regulation of muscle alternative splicing
Wbp5	NM_011712	0.28	Coding-3'UTR junction	Protein binding

a From NCBI Database.

## Abbreviations

DM: differentiation medium
DMEM: Dulbecco's Modified Eagle Medium
GM: growing medium
miRNA: microRNA
MSC: mouse satellite cells
PTB: polypyrimidine tract-binding protein
RISC: RNA-induced silencing complex
RISC-IP: RNA-induced silencing complex immunoprecipitation
