# Supplementary material for: MicroRNA-222 regulates muscle alternative splicing through Rbm24 during differentiation of skeletal muscle cells
Source: Cell Death Dis. 2016 Feb 4;7(2):e2086–. doi: 10.1038/cddis.2016.10 (PMC4849150; doi:10.1038/cddis.2016.10)
Supplement: Supplementary Table S3 [file cddis201610x3.docx]

Table S3. List of primers used for RT-PCR and cloning

| **Real-Time PCR Primers** | |
| --- | --- |
| Rbm24 F | TGGGTAAAGGCAGGCAGAAG |
| Rbm24 R | GTGGGGCAGGAGGAAAAGAA |
| Ahnak F | AGGGGATTGGCCTTGATGTG |
| Ahnak R | GCCCACCAGTAGCAATCTCA |
| D1Pas1 F | TGGAGGTAGCTATGGAGGCT |
| D1Pas1 R | TGGCAAAGCAGGCTCAGTTA |
| p27 F | GAGCAGTGTCCAGGGATGAG |
| p27 R | TCTGTTCTGTTGGCCCTTTT |
| Chrng F* | TGGTCACATGACTTTGAGCAGT |
| Chrng R* | GTGCTGGAAACCAACCCAGA |
| Myo10 F | ACGAGTCTGCTCACAAAGCA |
| Myo10 R | AGCCAAGAGACACTGCTTCC |
| Wbp5 F | GGCACCTGAGCAGTGATGAT |
| Wbp5 R | ATGGCTTCGGTTAGCCTTCC |
| NACA F | TACAGAGCAGGAGTTGCCAC |
| NACA R | CTAACTGTGCTTGCTGAGAC |
| skmNACA R | GCAGTTTCAGCTGTTATGGG |
| **PCR Primers** | |
| Coro6 F | TCATCATCTGGAATGTGGGC |
| Coro6 R | GTACCGAATGCTACTGTCAC |
| Fxr1 F | TCTCACCACAGTACTAACCG |
| Fxr1 R | TAGCCAAAGTTTCCCTTGGG |
| Chrng3UTR F | TGGTCACATGACTTTGAGCAGT |
| **PCR Primers for cloning in pMIR-REPORT** | |
| Rbm24 1 wt F | CTAGTGGATCCCTAGTTGAACCAGGGAATACGGAGTGAGCAATATGTAGCTTGAATTACGCGTAAAAGTA |
| Rbm24 1 wt R | AGCTTACTTTTACGCGTAATTCAAGCTACATATTGCTCACTCCGTATTCCCTGGTTCAACTAGGGATCCA |
| Rbm24 1 mut F | CTAGTGGATCCCTAGTTGAACCAGGGAATACGGAGTGAGCAATTTGAATTACGCGTAAAAGTA |
| Rbm24 1 mut R | AGCTTACTTTTACGCGTAATTCAAATTGCTCACTCCGTATTCCCTGGTTCAACTAGGGATCCA |
| Rbm24 2 wt F | CTAGTGGATCCATCTCATGGCATAAGTTATTCAGTAGGTCTAGATGTAGCATATTAAATATTAACCTATA |
| Rbm24 2 wt R | AGCTTATAGGTTAATATTTAATATGCTACATCTAGACCTACTGAATAACTTATGCCATGAGATGGATCCA |
| Rbm24 2 mut F | CTAGTGGATCCATCTCATGGCATAAGTTATTCAGTAGGTCTAGATATTAAATATTAACCTATA |
| Rbm24 2 mut R | AGCTTATAGGTTAATATTTAATATCTAGACCTACTGAATAACTTATGCCATGAGATGGATCCA |
| Ahnak1 wt F | CTAGTGGATCCCTGTAGGATTTCTATGGCAGATGTAGACTTAAATGTAGCTGCACCTAAAGGGAAAGGGA |
| Ahnak wt R | AGCTTCCCTTTCCCTTTAGGTGCAGCTACATTTAAGTCTACATCTGCCATAGAAATCCTACAGGGATCCA |
| Ahnak mut F | CTAGTGGATCCCTGTAGGATTTCTATGGCAGATGTAGACTTAATGCACCTAAAGGGAAAGGGA |
| Ahnak mut R | AGCTTCCCTTTCCCTTTAGGTGCATTAAGTCTACATCTGCCATAGAAATCCTACAGGGATCCA |
| **PCR Primers for cloning in pGL3-Promoter** | |
| Rbm24 3UTR F | TATACTAGTCTGCCTTTTCCAAGTTAGA |
| Rbm24 3UTR R | TATACTAGTCGAACAGTGCACATATGAAT |
| **PCR Primers for cloning in pCR2.1** | |
| Rbm24 cds F | TATACCGGTCCAATGCACACCACCCAGAAGG |
| Rbm24 cds R | TATGTCGACCTACTGCATTCGGTCTGTC |

Asterisks (*) indicate primers also used in conventional PCR experiments
