# Supplementary material for: MicroRNA-222 regulates muscle alternative splicing through Rbm24 during differentiation of skeletal muscle cells
Source: Cell Death Dis. 2016 Feb 4;7(2):e2086–. doi: 10.1038/cddis.2016.10 (PMC4849150; doi:10.1038/cddis.2016.10)
Supplement: Supplementary Figures [file cddis201610x4.pdf]

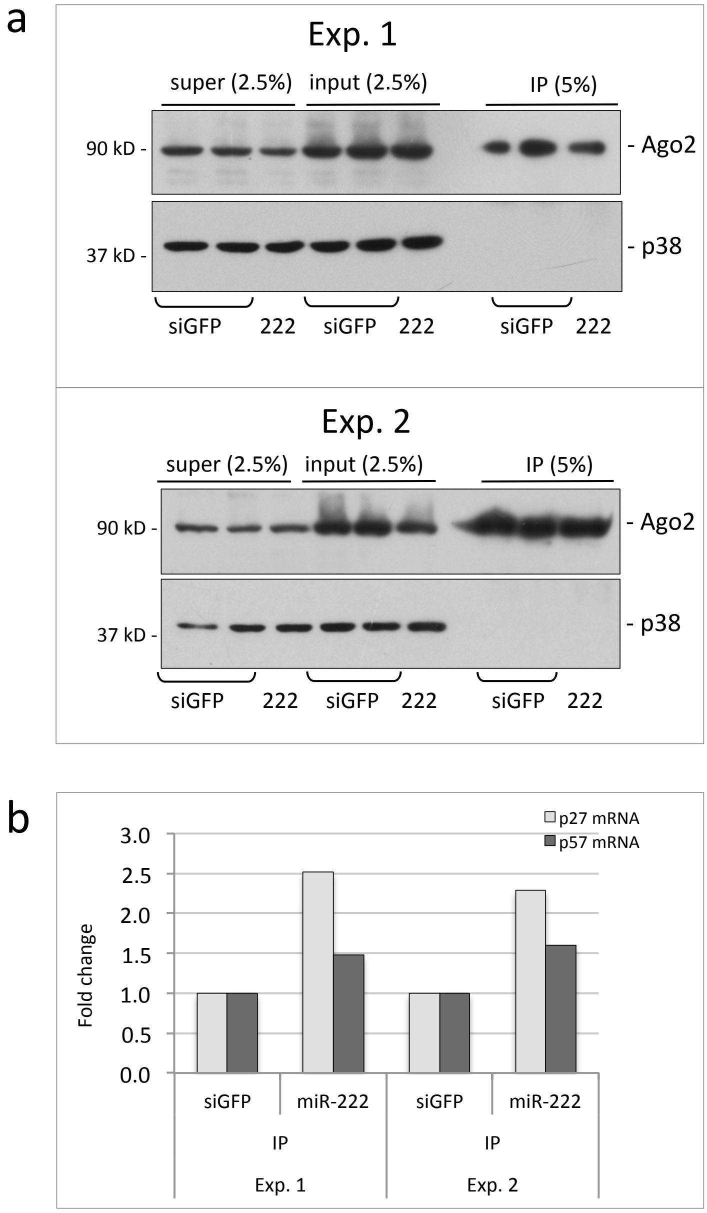

Figure S1 RISC-IP analysis. (**a**) Western blot of immunoprecipitated Ago2 protein (IP) compared to total Ago2 before IP (input) and to that remaining in the supernatant after IP (super) from MSC transfected with miR-222 mimics and control siGFP duplexes, in two independent experiments. p38 protein is shown as loading control. Percent of proteins analyzed with respect to total proteins for each fraction is indicated. (**b**) qPCR analysis of the miR-222 target mRNAs p27 and p57, normalized to GAPDH mRNA, in miR-222-IPs compared to siGFP-IPs shown in (**a**).

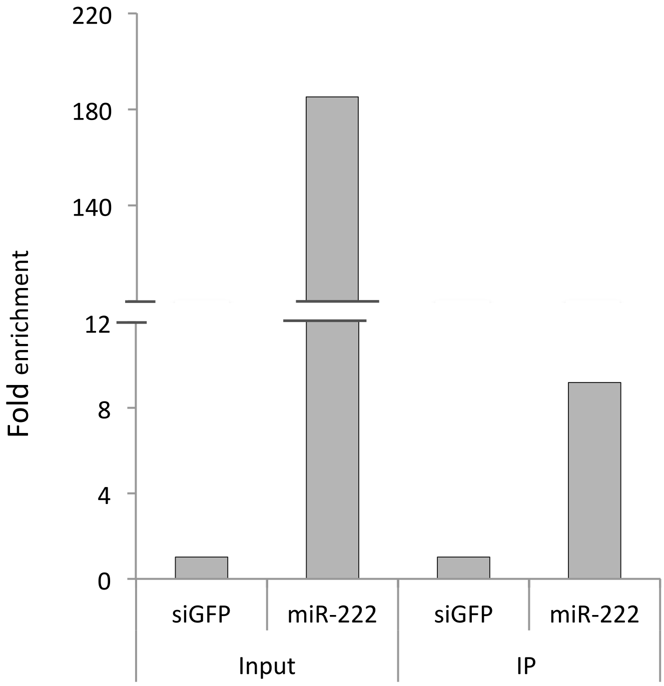

Figure S2 Analysis of miR-222 enrichment in Ago2 immunoprecipitates. qPCR quantitation of miR-222 levels, normalized to miR-16 levels, in Input and IP from MSC transfected with control siGFP or miR-222 mimics. A representative experiments is shown.

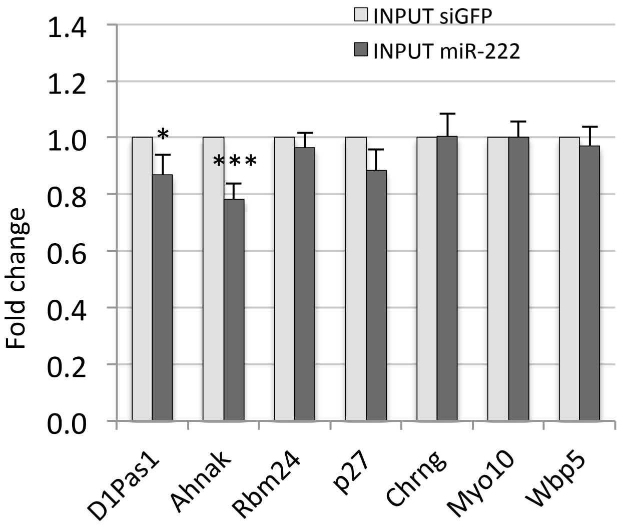

Figure S3 Analysis of miR-222 validated targets in input RNAs before RISC-IP. MSC myocytes, kept in DM for 30h, were transfected with 20nM miR-222 mimic and control siGFP and, after 16h, processed for RISC-IP. RNA extracted from the inputs were analyzed by qPCR for the expression of miR-222 mRNA targets. p27 mRNA is included for comparison. Values were normalized for GAPDH mRNA and the expression level of each mRNA is indicated as fold change in INPUT-miR-222 *versus* INPUT-siGFP, referred as 1. The error bars represent the average ± S.E. ($n \geq 3$; *P<0.05; ***P <0.001).

a

b

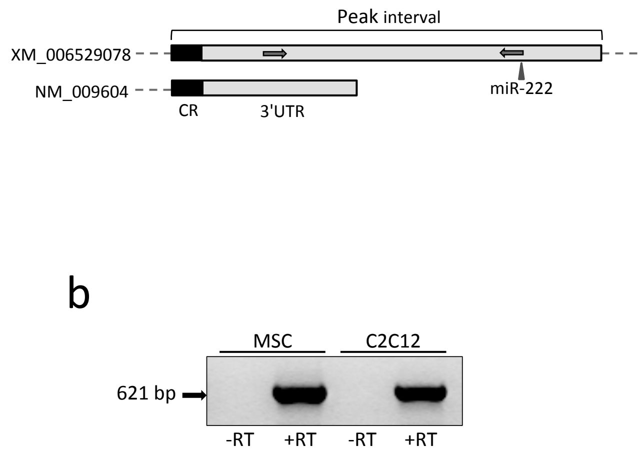

Figure S4 Analysis of a predicted Chrng mRNA isoform containing a miR-222 binding site. (**a**) Schematic representation of the the 3’UTRs downstream to the coding regions (CR) of the Chrng transcripts identified by the respective references. Arrows indicate the position of the primers used for amplification of a 621 bp fragment within the peak interval of miR-222 target site containing transcript, identified by RNA sequencing. (**b**) RT-PCR amplification +/- reverse transcription (RT) of RNA extracted from differentiated MSC and C2C12 myoblasts.

a

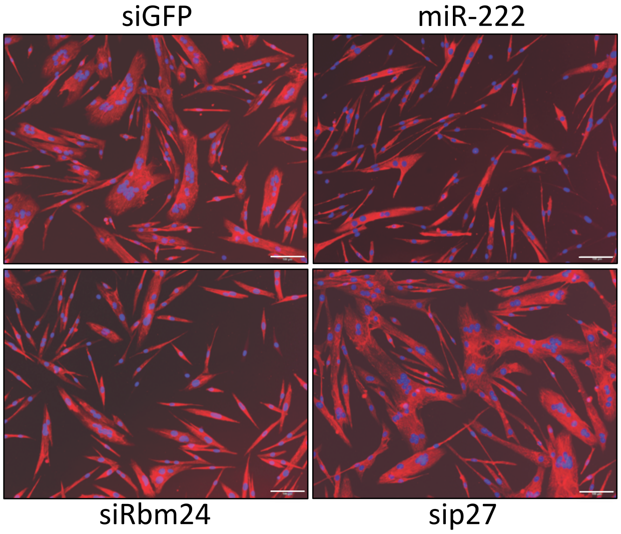

b

	% fusion	% MHC$^+$ cells
**siGFP**	**73.6** ± 4.4	**93.9** ± 3.0
**miR-222**	**36.9** ± 7.1	**96.4** ± 1.6
**siRbm24**	**50.2** ± 6.6	**99.6** ± 0.6
**sip27**	**73.9** ± 2.3	**95.4** ± 3.0

c

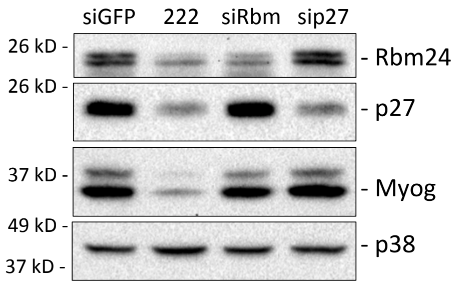

1.0	0.16	0.26	1.00	Rbm24/p38
1.0	0.15	0.95	0.23	p27/p38
1.0	0.06	0.70	1.08	Myog/p38

d

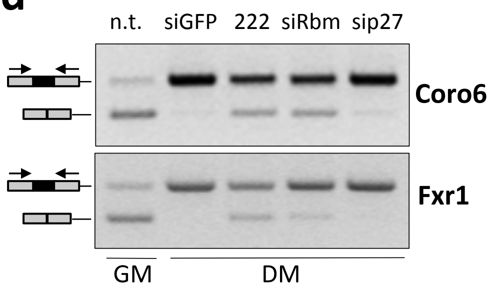

Figure S5 (**a**) MSC myoblasts transfected in GM with siGFP, miR-222, siRbm24 or sip27 duplex RNAs were shifted to DM for 2 days. Cells were subjected to immunofluorescence with antibodies specific for skeletal Myosin Heavy Chain (MHC) and nuclei were counterstained with Hoechst dye. Scale bar: 100 μm. (**b**) Quantitation of fusion and MHC positive (MHC$^+$) cells in DM in at least three independent experiments (averages ± S.E.) is shown in the table. (**c**) MSC myoblasts transfected as above were analyzed after 24 h in DM for expression of Rbm24, p27, and Myogenin proteins by Western blot. The table shows a quantification of the expression of Rbm24, p27 and Myogenin proteins normalized to p38, relative to control siGFP, referred as 1. (**d**) RNAs from parallel cell cultures were analyzed by semi-quantitative RT-PCR to determine splicing efficiency of muscular isoforms of Coro6 and Fxr1 transcripts.
